# Characterization of the complete mitochondrial genome of *Antheraea pernyi* strain Luhong

**DOI:** 10.1080/23802359.2021.1914226

**Published:** 2021-09-09

**Authors:** Ying Sun, Mao-Hua Pan, Ru-Bing Bai, Jing Li, Yue Zhang, Jin-Chen Yang, Jia-Qing Wang, Ming-Gang Ji, Yan-Qun Liu

**Affiliations:** a College of Life Engineering, Shenyang Institute of Technology, Fushun Liaoning, China; b Sericultural Institute of Henan Province, Zhengzhou, China; c Sericultural Institute of Liaoning Province, Fengcheng, China; d College of Bioscience and Biotechnology, Shenyang Agricultural University, Shenyang, China

**Keywords:** *Antheraea pernyi*, Luhong, mitochondrial genome

## Abstract

The present study for the first time describes the complete mitochondrial (mt) genome of *Antheraea pernyi* Guérin-Méneville 1855 strain Luhong, a genetic lethal mutant exhibiting especially red skin color. The mt genome is 15,563 bp in length that is the smallest among the sequenced *A. pernyi* inbred strains. This genome displays an identical genomic component and gene order to other six known *A. pernyi* mt genomes. The mt genome-based phylogenetic analysis clustered Luhong with four strains exhibiting yellow skin color, consistent with the traditional view that all of them belonged to the yellow blood lineage.

Chinese oak silkmoth, *Antheraea pernyi* Guérin-Méneville 1855 is not only an edible insect for human food but also a traditional silk-producing insect. It has already been used as an ornamental insect for agricultural tourism in China due to its colorful larvae including yellow-cyan, white, blue, and yellow. Up to now, more than 100 inbred strains with diverse agronomic characters have been developed. Luhong is one such strain exhibiting especially red skin color used for agricultural tourism. The homozygotes of this strain (∼25%) are genetically lethal during embryonic development. The remaining individuals exhibit yellow skin color from 2nd to 4th instar stages, but in the 5th instar stage two-thirds become red and one-thirds maintain yellow (Liu et al. 1999). The founder individuals of this strain with red skin color was found from a local strain ‘Luhuang_1’ exhibiting yellow skin color in Lushan of Henan Province (Sericultural Institute of Liaoning Province 1994). The present study determines the mitochondrial (mt) genome of this strain for the first time to understand the basic genetic information.

Specimens of the inbred strain Luhong were provided by the Sericultural Institute of Henan Province (N33°26′; E112°44′), Zhengzhou, China. Specimens used in this study were preserved in 95% ethanol and deposited at the Department of Sericulture, Shenyang Agricultural University (Yan-Qun Liu, liuyanqun@syau.edu.cn) under the voucher number OAK_SILKWORM_LUHONG_01. The total genomic DNA was extracted from a single individual. The long PCR amplification, sequence determination and genomic annotation were done as previously described for Qinghuang_1 (Li et al. 2020). The mt genome was manually annotated by comparing with the published mt genomes of *A. pernyi*.

The full mt genome of strain Luhong is 15,563 bp in length and also contains a typical insect mt gene set including 13 protein-coding genes (PCGs), 22 tRNA genes, and two ribosomal RNA genes. The genomic component and gene order are identical to other six known *A. pernyi* mt genomes as well as known Bombycidea species. The A + T-rich region is 552 bp in size harboring an 18 bp poly-T stretch preceded by the ATAGA motif but a 19 bp poly-T stretch in the other six known *A. pernyi* mt genomes. In the A + T-rich region, a 16 bp poly-A element upstream *tRNA^Met^* and intermittently interrupted by T and G is also present for Luhong; however, a 16 bp poly-A element intermittently interrupted by G occurs in two strains (Yu_7 and 731) and a 15 bp poly-A element intermittently interrupted by G occurs in four strains (Yu_6, Yuzao_1, Qing_6 and Qinghuang_1). Comparison analysis indicated that length variation of the two conserved structural features has occurred among strains of *A. pernyi*.

The intraspecific phylogenetic relationships of seven *A. pernyi* strains available to date were assessed based on the nucleotide sequence of mt genomes with maximum-likelihood and Bayesian inference methods under GTR + G + I nucleotide substitution model (Figure 1). Three *Antheraea* species *A. yamamai*, *A. frithi,* and *A. assamensis* were also included and two non-*Antheraea* species served as outgroups. Bayesian inference and maximum-likelihood methods were conducted using MrBayes ver. 3.1.2 and IQ-TREE 1.6.12, respectively (Huelsenbeck and Ronquist 2001; Nguyen et al. 2015). The two phylogenetic trees obtained are congruent with strong support for almost all nodes. The phylogenetic analyses recovered two groups for the seven *A. pernyi* strains used. Group one contained Qing_6 and Qinghuang_1, both of them displaying yellow-cyan skin color were derived from ‘Aiyang’ population of Liaoning Province. Another group contained five strains, all of which showing yellow skin color were derived from ‘Lushan’ population of Henan Province. The mt genome-based phylogenetic analysis clustered Luhong with the other four strains with yellow skin color, consistent with their breeding histories.

**Figure 1.**
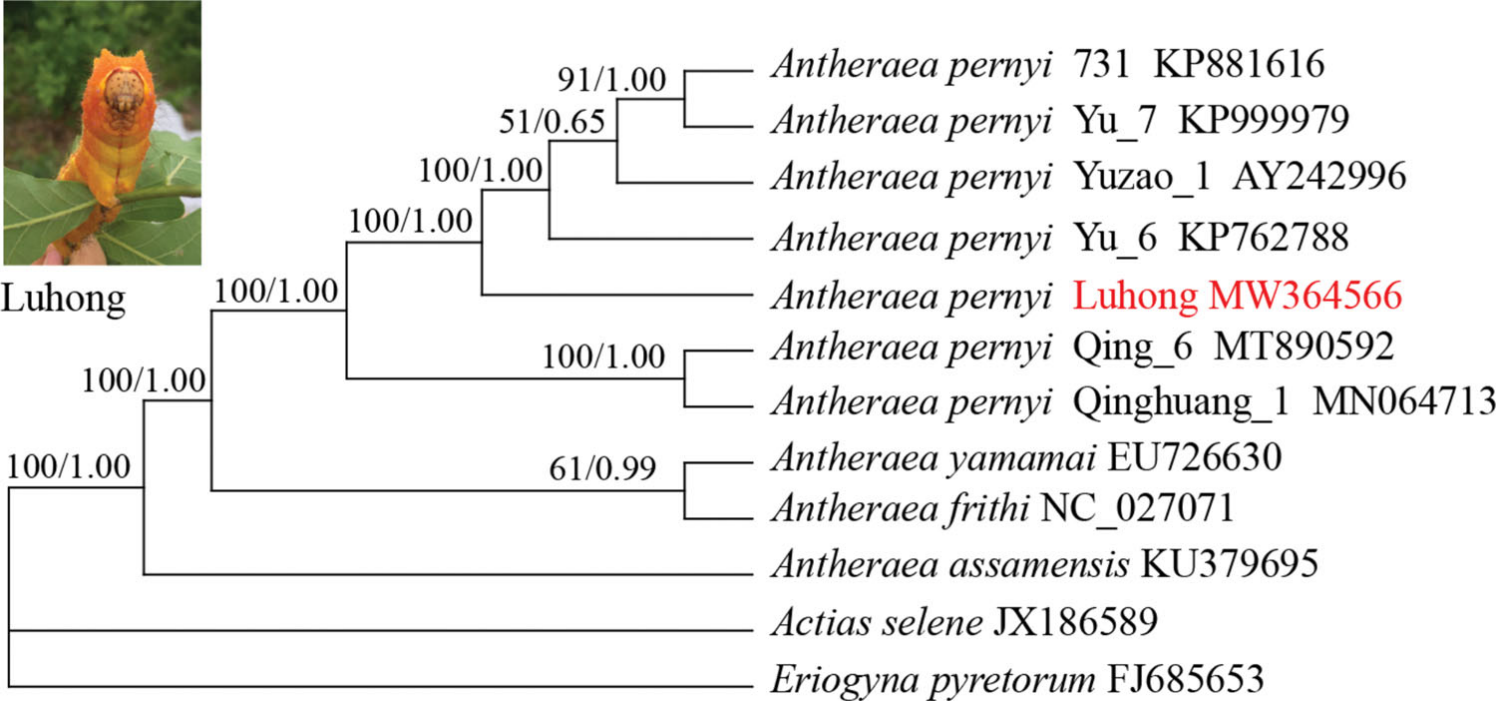
Phylogenetic trees inferred from full mitochondrial genomes using maximum-likelihood and Bayesian inference methods under GTR + G + I model. The bootstrap values (former) and posterior probability (latter) values are indicated at the nodes. GenBank accession numbers are listed following the name of each species or strain.

## Data Availability

The genome sequence data that support the findings of this study are openly available in GenBank of NCBI at (https://www.ncbi.nlm.nih.gov/) under the accession no. MW364566. The associated BioProject, SRA, and Bio-Sample numbers are PRJNA695117, SRR13558698, and SAMN17601314, respectively.
